# Post-traumatic stress symptoms and professional quality of life among healthcare professionals working in COVID departments

**DOI:** 10.1192/j.eurpsy.2022.1378

**Published:** 2022-09-01

**Authors:** O. Elleuch, R. Feki, N. Smaoui, I. Gassara, M. Maalej, S. Omri, N. Charfi, L. Zouari, J. Ben Thabet, M. Maalej

**Affiliations:** 1 Hedi Chaker Hospital, Sfax, Psychiatry C, Sfax, Tunisia; 2 Hedi Chaker University Hospital, Psychiatry C, Sfax, Tunisia; 3 Hedi Chaker university hospital, Psychiatry C, Sfax, Tunisia

**Keywords:** post-traumatic stress, Porfessional quality of Life, Healthcare professionals, Covid-19

## Abstract

**Introduction:**

The COVID pandemic had a heavy impact on the mental health of people in general and healthcare professionals in particular.

**Objectives:**

Our study aimed to examine the the prevalence of post-traumatic stress symptoms among healthcare professionals working in COVID departments, and assess their professional quality of life.

**Methods:**

Our sample consisted of 23 healthcare professionals who are working in the COVID departments of the Hospitals of Sfax. We collected their sociodemographic data, their medical history and COVID-related details. Their mental health was assessed by the Impact of Event scale (IES-R) and the professional quality of life scale (ProQOL-5)

**Results:**

The sex ratio in our study was 17:6, with a mean age of 31.79 years. They carried out 5.43 nightshifts per month, 57 hours of work per week including 27.38 hours of direct contact with COVID positive patients. A rate of 21.74% of the patients had a high IES-R score, indicating severe post traumatic stress symptoms. As for the subscales of the professional quality of life score, 21.73% of the participants had a low compassion satisfaction score, 65.21% of the participants had a moderate one and 13% had a high one. A rate of 91.3% of the participants had a moderate burnout score, the mean was 29.39. The secondary traumatization score was low in 26% of the cases, moderate in 60.86%, high in 13% and the mean was 27.91.

**Conclusions:**

COVID healthcare professionals are at a relatively high risk of developing burnout and post-traumatic stress symptoms.

**Disclosure:**

No significant relationships.

